# The “Fostering Changes” Parent Training Programme for Foster Carers: A Feasibility Study of the German Version

**DOI:** 10.3390/children13010057

**Published:** 2025-12-30

**Authors:** Judith Bürzle, Sarah Degen, Christian J. Bachmann

**Affiliations:** Department of Child and Adolescent Psychiatry, Ulm University Medical Center, Steinhövelstr. 5, 89075 Ulm, Germany; judith_buerzle@yahoo.de (J.B.);

**Keywords:** attachment, foster carers, foster children, parent training, parenting, psychopathology, youth welfare services

## Abstract

**Highlights:**

**What are the main findings?**

- This study found that the German-translated version of the *Fostering Changes* programme was acceptable to foster parents in Germany, as demonstrated by high participant retention.
- Participation in *Fostering Changes* was associated with effects similar to those found in UK studies with regard to child behaviour, child psychopathology, parenting skills, and the relationship between foster parents and foster child.

**What are the implications of the main findings?**

- The results of this study indicate that the German version of *Fostering Changes* in its current form can be used in Germany without further adaptation and can be delivered effectively without the direct involvement of its original developers.
- The German version of *Fostering Changes* has the potential to be an effective intervention for both foster carers and foster children, especially with regard to child behaviour and child psychopathology.

**Abstract:**

**Background:** Foster children exhibit higher rates of psychiatric and physical disorders than children living with their biological families. This places a high burden on the parenting skills of foster parents and potentially increases the risk of placement failure. One possibility to increase foster carers’ parenting skills and to reduce child problems is through parent training. In this study, the feasibility and effectiveness of the German-translated version of *Fostering Changes*, a parent training programme for foster parents, was investigated. The aims of *Fostering Changes* are the reduction in child behavioural problems, supporting children’s affect regulation, and improving the quality of the foster parent–child relationship through the promotion of foster parents’ sensitivity and parenting skills. **Methods:** We conducted six *Fostering Changes* courses in 2022 and 2023, with a total of 33 foster carers (i.e., foster parents) participating. Child behavioural problems (Carer-Defined Problems Scale; primary outcome), child psychopathology (Strengths and Difficulties Questionnaire), carer–child relationship quality (Child Relationship Behavior Inventory, Quality of Attachment Relationship Questionnaire), foster carers’ stress (Parental Stress Scale), and foster carers’ parenting strategies (Parenting Scale) were assessed at the start (t0) and end of each course (t1) and three months after course completion (t2). To examine the effect of training participation, mixed linear models and generalised estimating equations were applied. Additionally, effect sizes (Cohen’s *d)* were calculated. **Results:** When comparing t0 with t1 scores, there was a significant reduction in child behavioural problems (*d* = 1.87) and child psychopathology (*d* = 0.70), and improvement in foster carers’ parenting skills (*d* = 0.76) and the quality of the foster parent–child relationship (CRBI: *d* = 0.77, QUARQ: *d* = 0.72). Effect sizes for changes in the abovementioned variables between t0 and t2 were also moderate to large, with the exception of child psychopathology (*d* = 0.44). **Conclusions:** The results of this feasibility study, which is the first trial of *Fostering Changes* outside the UK, suggest that the German version of *Fostering Changes* could be an effective intervention for foster families. The largely comparable results for the periods t0–t1 and t0–t2 suggest constancy of the observed changes three months after course completion. Trial registration: DRKS-ID: DRKS00029014; date of registration: 23 May 2022.

## 1. Introduction

Foster children can be considered as a high-risk group. Between 50% of preschool [1] and 80% of foster children between 11 and 18 years [2] have experienced at least one traumatic event. Moreover, around 40% of preschool foster children have developmental delays [3]. Also, foster children have higher rates of foetal alcohol spectrum disorder (FASD) than in the general population [4]. In addition, the prevalence of mental disorders in foster children is also higher than the general population [5]. This constellation can produce high demands on foster carers, which may even lead to placement breakdowns [6]. Due to the demanding nature of the fostering role, foster carers experience high levels of secondary traumatic stress, burnout, and compassion fatigue, which may reduce caregiving satisfaction, impair care quality, and increase the risk of placement breakdowns [7,8,9,10].

On the other hand, successful placements in foster families can reduce internalising and externalising mental health symptoms more effectively than placements in residential care [11]. Also, fewer placement disruptions and staying longer within the same foster family can constitute a protective factor for child mental health [12] and school attendance [13], and therefore on future educational prospects. Furthermore, foster children can form secure attachments with their foster parents, even if they are insecurely attached to their biological parents [14].

In light of these positive effects, foster parents appear to be a promising target group for measures to prevent placement breakdowns and to promote child development. A meta-analysis by Schoemaker et al. [15] showed that interventions for foster parents can increase parental sensitivity and reduce dysfunctional parenting strategies, parental stress, and behavioural problems of foster children in the range of moderate to large effect sizes.

The German foster care system has some specific characteristics: In Germany, more children and adolescents live in residential care than in foster families. In 2023, approximately 87,000 children and adolescents lived with foster families, compared to around 128,000 placed in residential care [16]. This distribution contrasts with other European countries, such as the United Kingdom (UK), where roughly three quarters of looked-after children live in foster families [17]. Foster care placements in Germany are often based on cooperative arrangements involving birth parents, foster carers, and youth welfare offices [17]. Consequently, foster parents often do not hold child custody, which may result in restricted access to information about the foster child, limited decision-making authority for the foster child, and uncertainty regarding the foster child’s long-term placement [18]. In cases of reunification attempts by biological parents, who retain custody, German family courts must balance parental rights against the child’s best interests and rights. Foster parents in Germany are recruited, assessed, prepared, and supported by staff from the local youth welfare offices, although these tasks may be delegated to private or non-profit child and youth welfare organisations. However, no nationwide standards exist regarding the extent of or obligation to preplacement and ongoing training for foster parents. In some regions, there is no obligation for further training at all. By contrast, the foster care system in the UK, which has been formally regulated since the Adoption of Children Act of 1926, requires a minimum of 24 h of training within the first year of a placement, followed by an additional 24 h every three years [17].

Also, the evidence base for parent trainings used for foster carers in Germany is scarce, especially for foster children over the age of two up to and including school age. Regarding interventions for foster parents in Germany, there are only two published studies that have systematically investigated their effectiveness: Job et al. [19] tested the effectiveness of the “Triple P for Carers” programme in a randomised-controlled trial (RCT; *N* = 81 foster families with 87 foster children aged 2–7 years) and found no significant difference between the two groups on any of the studied outcomes (e.g., parenting style, quality of foster parent–child relationship, child behavioural problems). Another uncontrolled trial evaluated the “Attachment and Biobehavioural Catch-Up” programme (*N* = 34 foster families with foster children aged 6–24 months) [20]. Post-training, the foster carers behaved significant more sensitively and showed more emotional warmth in a semi-structured play situation with their foster child.

A parenting programme designed for this age group is *Fostering Changes*. It is a manualised training for foster parents of children aged 2–11 years with emotional and/or behavioural problems. Yet, a psychiatric diagnosis in the foster child is not required for participation, in order to keep the barriers for participation low. The training was developed by clinicians from the National Adoption and Fostering Clinic at the Maudsley Hospital in London [21]. The training is based on social learning theory as well as on results from trauma research and attachment theory and takes the special nature of the fostering relationship in consideration. Each course consists of 12 weekly three-hour group sessions. The aims of *Fostering Changes* are reducing children’s problem behaviour, supporting children’s affect regulation, and improving the quality of the parent–child relationship through the promotion of foster parents’ sensitivity and parenting skills. Based on social learning theory, course facilitators play a crucial role by modelling in their interactions with foster parents how the foster carers might relate to their foster children at home. This includes, for example, praising participation in role-plays or responding empathetically to stressful experiences shared by carers. At the same time, the goal is to create a positive learning environment based on a culture of openness, enabling foster carers to share their own uncertainties and failures within the group. In line with foster carers’ feedback [22] and principles of adult learning theory, brief theoretical lectures alternate with role-play to practice parenting strategies and structured exchange to engage in self-reflection on one’s own biography. Carers are also encouraged to apply the strategies at home with their foster child and share their experiences in the next session (for detailed information about the content of *Fostering Changes*, see Supplementary Table S1). Early research on parent training already highlighted role-play as a key approach, as it enables parents to translate abstract principles into concrete strategies and to actively practice new interaction patterns in a safe and supportive setting with immediate feedback. Through guided role-play and structured reflection, parents directly experience helpful vs. unhelpful behaviours and associated emotions, rather than reflecting on them solely at an abstract level [23].

Pallett et al. [22] and Warman et al. [24] investigated the effectiveness of *Fostering Changes* in uncontrolled trials in the UK, which yielded promising results (Table 1). Following this, Briskman et al. [25] conducted an RCT of *Fostering Changes* in London, using a waiting list control group, and reaching small to large effect sizes in terms of foster children’s mental health and behaviour. Moody et al. [26] established an RCT of *Fostering Changes* in Wales. In contrast to previous results, solely child psychopathology decreased significantly post-intervention. At 9-month follow-up, no differences between intervention and control group were found.

## 2. Materials and Methods

This feasibility study aimed to investigate whether the translated version of *Fostering Changes*, originally developed and well-proven in the UK, would also be effective in Germany in terms of increasing the quality of the foster parent–foster child relationship, decreasing parental stress and harsh parenting and reducing behavioural problems of foster children.

### 2.1. Participants

All foster parents from two districts in Southern Germany (East Württemberg, Bavarian-Swabia) were eligible to participate in the trial. Inclusion criteria were (a) foster parents’ fluency in German (for participation in group activities), (b) caring for at least one foster child aged 2–11 years for the full duration of the course, and (c) no diagnosed intellectual disability in the foster child. When both foster parents of the same foster family wished to participate, they were asked to select two different foster children as their particular index child, if they cared for more than one foster child who met the inclusion criteria.

### 2.2. Recruitment

*Fostering Changes* courses were offered in two cities in East Württemberg. All adjacent youth welfare services were informed via mail about the study and the opportunity for foster carers to participate for free. Also, child and adolescent psychiatrists and outpatient clinics, paediatric centres and counselling services for parents of preschool children with special needs in the region were contacted by mail.

### 2.3. Trial Design

We conducted an uncontrolled trial, using a mixed methods design with method triangulation. Quantitative data were collected using questionnaires at three time points, while qualitative data were collected through group interviews conducted two months after the end of the course. In this article, only the quantitative data are reported.

### 2.4. Outcome Variables and Measures

Prior to the course, an initial home visit, or alternatively a video call, was offered to interested foster carers. The initial visit usually lasted one to two hours and included information about the course. Socio-demographic characteristics of foster children and carers were collected during this visit, using a semi-structured interview guideline (based on Bachmann et al. [21]). Evaluation questionnaires were completed in paper–pencil format during the initial session (t0; the first of twelve weekly three-hour sessions) and the final session (t1; session 12 of 12, 12–14 weeks after t0) of the course. Three months after the end of the course (t2) questionnaires were sent to the participants by mail.

To ensure comparability with the existing studies on *Fostering Changes*, similar outcome variables and measures were selected. The primary outcome was child problem behaviour; secondary outcomes were child psychopathology, parenting style, parental stress level, and quality of the foster child–foster parent relationship.

#### 2.4.1. Child Problem Behaviour

Using the *Carer-Defined Problems Scale* (CDPS; [25]), foster parents were asked to briefly name the three most severe problem behaviours of their foster child. Afterwards, they had to rate the extent of these difficulties on a visual analogue scale, with higher values indicating a higher level of difficulties (*range* of the total score: 0–30). The CDPS instructions were translated into German by the study authors.

#### 2.4.2. Child Psychopathology

The *Strengths and Difficulties Questionnaire* (SDQ; [27]) consists of 25 items which can be rated on a three-point Likert scale. The items can be assigned to five domains (Emotional Problems, Conduct Problems, Hyperactivity/Inattention, Peer Problems, and Prosocial Behaviour). A Total Difficulties score (SDQ-total) can be derived from the first four domains mentioned above, with higher scores indicating more psychopathological symptom severity (*range:* 0–40). For the German parent version of the SDQ, Klasen et al. [28] reported very good criterion validity, with correlations between the SDQ-total score and the German version of the *Child Behavior Checklist* ranging from 0.78 to 0.82. In addition, Rothenberger et al. [29] demonstrated good internal consistency for the SDQ-total score of the German parent version, based on a sample of the general population with Cronbach’s α = 0.82. In the present study, internal consistency of the SDQ-total scale ranged from acceptable to good (Cronbach’s α = 0.72–0.82) depending on the assessment time.

#### 2.4.3. Parenting Style

The German short version of the *Parenting Scale* (PS-S; [30]) employs 13 items to assess the three parenting dimensions of *Overreactivity*, *Laxness*, and *Monitoring*, using a seven-point Likert scale for each item. A mean value score (*range*: 1–7) is calculated based on the total score of all items divided by the total number of items, with higher mean value scores indicating greater use of dysfunctional parenting strategies. Miller [30] showed an acceptable internal consistency of α = 0.76 for the short version of the PS within a sample of parents of children between 3 and 6 years recruited in kindergartens. The short and long version of the PS [31] correlated highly with each other [30]. In the present study, the internal consistency of the PS-S ranged from acceptable to good (Cronbach’s α = 0.75–0.86).

#### 2.4.4. Parental Stress

The German version [32] of the *Parental Stress Scale* (PSS; [33]) uses 18 items scored on a five-point Likert scale to assess the individual experience of stress with regard to one’s own parenting. A higher total score (*range*: 18–90) indicates a higher level of stress. For the German version of the questionnaire, good internal consistency was demonstrated by Kölch et al. [34] within a sample of the general population (McDonalds ω = 0.85), which is comparable to the English version (Cronbach’s α = 0.83 (33)). In the present study, the internal consistency of the PSS ranged from acceptable to good (Cronbach’s α = 0.72–0.86).

#### 2.4.5. Foster Parent–Foster Child Relationship

Briskman et al. [25] designed the *Quality of Attachment Relationship Questionnaire* (QUARQ) consisting of 16 items. The questionnaire explores whether the foster child is able to show its feelings and ask for support from the foster parents in stressful situations and whether the foster carer can understand the child’s feelings. A higher general score (*range*: 0–64) indicates a closer relationship between foster parent and child. The QUARQ was translated into German by the authors. In the present study, the internal consistency of the QUARQ, as measured by Cronbach’s alpha, was calculated for each of the three measurement points. Item 13 was excluded from the reliability analysis because it showed no variance in the sample at follow-up. Reliability coefficients ranged from good to excellent (Cronbach’s α = 0.83–0.91).

There are currently no systematic validation studies available for the QUARQ; therefore, the *Child Relationship Behavior Inventory* (CRBI; [35]) was additionally employed. Based on 30 items, the frequency of relationship-promoting (*Child Relationship Development Questionnaire*, CRDQ Frequency Scale, total score range: 14–98) and of relationship-damaging behaviours (*Child Relationship Checklist*, CRC Frequency Scale, *range*: 16–112) of the foster child is investigated from the foster parents’ perspective. By dividing the total score of the CRDQ Frequency Scale by the total score of the CRC Frequency Scale, an individual CRBI ratio score can be calculated. Briegel et al. [35] showed good internal consistency (Cronbach’s α = 0.86–0.90) for the CRDQ and CRC Frequency Scale, with a sample of parents of children between 2 and 10 years from day care centres and elementary schools. In the present study, reliability coefficients of the CRDQ Frequency Scale ranged from acceptable to good (Cronbach’s α = 0.78–0.85); those of the CRC Frequency Scale can be considered as good (Cronbach’s α = 0.86–0.89).

### 2.5. Intervention and Conditions of Implementation

From September 2022 to July 2023, a total of six *Fostering Changes* courses were offered by two course facilitators, using the German version of the *Fostering Changes* manual (for detailed information, please refer to Bachmann et al. [36]). A summary of the session topics can be found in Supplementary Table S1. Participants who were unable to attend a session were sent the session materials and received a telephone call for a short briefing from a course facilitator. To ensure treatment fidelity, the course facilitators completed post-session checklists of intervention content, methods, and their approach towards the participants. Additionally, they received regular supervision sessions from two experienced *Fostering Changes* trainers from the UK regarding the implementation of the course manual, and for reflecting their approach towards specific participants.

## 3. Data Analysis

Missing data were examined at the item and questionnaire level. As only 3.4% of item-level data and 3% of total questionnaire scores were missing, single imputation was used for isolated items by replacing missing item values with the mean of the respective scale or total score of the specific questionnaire. This procedure was chosen as—apart from three datasets—only 1 to 7 of 315 possible items per dataset were missing.

The data of this feasibility study is clustered, as the same participants provided repeated measures across three time points. Also, two foster parents independently rated the same foster child as the index child of the intervention, and one foster mother participated twice, each time with a different index child.

For completely missing datasets (three datasets at t2), no imputation was performed. To examine the effects of participation in a *Fostering Changes* course for the whole sample, mixed linear models (MLM) and generalised estimating equations (GEE) were used, as these procedures are able to account for repeated measures, the clustered data structure, and missing data. The three assessment points were included as an independent time variable tested for effects on the outcome variables. MLMs were applied to approximately continuous and normally distributed data. Normal distribution was tested with the Kolmogorov–Smirnov test; violations occurred for the QUARQ at t0 (*p* = 0.005) and t2 (*p* = 0.008), and for the CDPS total score at t0 (*p* = 0.004). These variables were analysed using GEEs for ordinal data, using cumulative logit link function, and robust covariance estimation (independent correlation structure, Fisher scoring, maximum likelihood).

Additionally, to facilitate comparability with other intervention studies, effect sizes (Cohen’s *d*) were computed, using the average of group variances as the standardisation method.

Multiple imputation was not applied because imputing t2 data based on results of earlier time points could have produced overly optimistic estimates, given the observed data tendency with improvement from t0 to t1, followed by stabilisation (means and standard deviations across the three assessments are provided in Supplementary Table S2).

All analyses were conducted using IBM SPSS Statistics (Versions 28 and 31) and Microsoft Excel 2019.

## 4. Results

### 4.1. Participants

Thirty-six foster parents received an initial visit (see Flowchart, Supplementary Figure S1). Of these, 33 foster carers met the inclusion criteria and participated in the *Fostering Changes* training. For the analysis, only data from foster parents who provided information for at least two time points was included (*N* = 32). For demographic information, see Table 2.

At course start, all foster parents had already received some support from local foster care agencies as part of the public youth welfare. This included, for example, preparatory courses for new foster carers (87.1%) and opportunities for an informal exchange with other foster families (71.0%). 41.9% of foster children had received support through social welfare agencies since their placement in the current foster family, e.g., school assistants for children with special needs. 48.4% of foster children had received or were still undergoing treatment by a child and adolescent psychiatrist or psychotherapist (for more detailed information, see Supplementary Table S3).

### 4.2. Attendance and Group Size

On average, the participating foster parents attended 10 out of 12 sessions (*M* = 10.2, *SD* = 1.9, *range*: 3–12), resulting in a mean attendance rate of 85.4%. Group sizes varied between 4 and 8 participants. The dropout rate was 6.1% (2 out of 33 participants).

### 4.3. Missing Data

Missing data accounted for 3.4% of all questionnaire items across all datasets and time points (350 out of 10,045 items). At the level of single datasets, with only three exceptions, between 1 and 7 out of 315 possible items were missing. Regarding total questionnaire scores (six questionnaires at three time points per dataset), 3% of values were missing (18 out of 594 possible scores). At the level of single datasets, total questionnaire scores were missing for three datasets. In all instances, the missing data occurred at t2, typically due to non-return of follow-up questionnaires, resulting in a complete absence of t2 data. To examine the mechanism of missing data, Little’s MCAR test was conducted. The test was non-significant, χ^2^ (12) = 13.25, *p* = 0.351, indicating that the data were missing completely at random (MCAR).

### 4.4. Effects of Fostering Changes

The statistical models employing the variable “time” yielded significant results for the following outcomes: child problem behaviour, child psychopathology, foster carer–foster child relationship, and foster carer parenting style (*p* < 0.001 or *p* = 0.003; for more statistical parameters, see Table 3). Although parenting stress experienced by foster parents decreased over time, this reduction was not statistically significant (*p* = 0.058; see Table 3).

From t0 to t1, there were large effect sizes for decrease in child problem behaviour, as measured by the CDPS (*d* = 1.87), and medium effect sizes for reduction in child psychopathology (SDQ-total: *d* = 0.70). Foster carers’ parenting skills (PS-S: *d* = 0.76) and the quality of the parent–child relationship (CRBI ratio: *d* = 0.77, QUARQ: *d* = 0.72) improved significantly in the range of moderate effect size. Effect sizes (Cohen’s *d*) for the outcomes are shown in Table 4.

When comparing t0 to t2, effect sizes were again in the moderate-to-large range, with the exception of child psychopathology (SDQ-total: *d* = 0.44). Consistently, comparisons of the results of t1 and t2 showed only very small effects (*d* = 0.01–0.09), except for child psychopathology (*d* = 0.21).

## 5. Discussion

At the level of the whole sample, participation in a *Fostering Changes* course was associated with significant reductions in child problem behaviour and psychopathology, as well as significant improvements in parenting skills and in the quality of the foster carer–child relationship. These effects were moderate to large.

Across measurement points, effect sizes were similar when comparing pre- to post-intervention and pre- to follow-up assessments—except for child psychopathology as an outcome variable, indicating that achieved improvements were largely maintained three months after the course participation.

Our results are in line with previous findings from *Fostering Changes* trials in England [22,24,25], with the effect sizes found in our feasibility study even exceeding those of the UK studies. This is a surprising finding, as effect sizes typically decline when interventions are implemented not by the programme developers themselves but by independent providers [37]. One reason for this finding might lie in the clinical and academic qualification level of the lead course facilitator, who was an experienced child and adolescent psychotherapist with an additional diploma in trauma-focused psychotherapy and clinical experience with children with attachment issues, while most UK *Fostering Changes* studies so far mainly employed social workers as course facilitators. Another factor that should be considered as an explanation for the difference in results between studies is treatment fidelity: In the current feasibility study, actions (e.g., post-session checklists and supervision session) were taken to ensure an implementation with high programme fidelity, which has shown to be associated with parental behaviour change and, in turn, child behaviour change [38,39,40,41]. In contrast, Moody et al. [26] did not report information on treatment fidelity for the *Fostering Changes* courses conducted as part of their trial in Wales.

The study had an attendance rate of 85.4%, and a rather low dropout rate of 6.1%, which is in line with the findings of Pallett et al. [22], who reported a dropout rate of 10% and an average attendance rate of 80%. Initial home visits within the *Fostering Changes* programme, which help foster parents to develop realistic expectations of the course, to identify personal relevance, and to create a connection to one of the course facilitators, as well as the supportive, positively focused, and validating approach of the course facilitators towards the participating foster parents, may serve as strategies to increase attendance rates at the first or subsequent appointments for parenting interventions and lessen premature termination [42].

Comparing the beginning of the course and the 3-month follow-up, we observed mainly similar effect sizes on most outcomes like comparing the start and end of the programme, with the exception of child psychopathology. Most of the improvement occurred between the start and end of the course, with little further change up to the follow-up assessment, suggesting short-term stability of the achieved changes. Future research should assess whether booster sessions after course participation might help to continue improvements, and whether the observed stability of changes holds up at a later follow-up assessment. In contrast, in the work of Moody et al. [26], at 9-month follow-up no significant effects were found for foster parents’ self-efficacy, foster parent–foster child relationship quality, or child psychopathology. However, comparability between trials is limited, as Moody et al. [26] used a control group in addition to the intervention group and calculations were based on the comparison of these two groups.

Interestingly, participation in a *Fostering Changes* course did not lead to a reduction in foster carers’ parental stress, despite the fact that, at baseline, the study sample reported higher levels of perceived parental stress (*M* = 44.42, *SD* = 9.19) than parents from the general population in a representative German sample assessed with the same instrument (*PSS*: *M* = 33.14, *SD* = 9.22; [34]). One explanation might be that implementing new family rules, adjusting one’s parenting style, trying new communication techniques, etc., as a consequence of attending a *Fostering Changes* course might at first lead to even more parental stress, with potential gains coming only with some delay, which cannot fully be measured within a 3-month follow-up period. This could explain why the results of this study contrast the existing literature: Lohaus et al. [43] showed that foster children’s externalising symptoms are the most important predictor of parental stress, whereby perceived social support—in their study, operationalised as the extent of partnership support—was not an additional explanatory factor. Another possible explanation relates to the choice of outcome measure. The measure used in this study (PSS) primarily captured parenting-related stress and may have been less sensitive to other relevant stress dimensions, such as secondary traumatic stress or compassion fatigue, which are commonly assessed using instruments such as the Professional Quality of Life (ProQOL). Consequently, certain intervention effects may not have been discovered, although *Fostering Changes* explicitly targets foster carers’ self-care and parenting competencies, which may positively influence secondary traumatic stress and compassion fatigue [7,8].

Although several studies have provided evidence supporting the effectiveness of *Fostering Changes* so far, it remains unclear which kind of foster parents and children benefit best from the programme. In an exploratory analysis of our dataset, foster child characteristics (age, sex, current placement duration, prior and ongoing psychotherapeutic or psychiatric treatment) emerged as significant influencing and moderating factors for a variety of outcomes (see Supplementary Table S4). On the foster parent level, attendance rate was a significant covariate in terms of child behavioural problems. Interestingly, foster parents’ level of experience or educational background did not play a significant role regarding outcomes. This might indicate that *Fostering Changes* is a programme universally suitable for foster parents of all backgrounds, and in all stages of their development as foster carers.

In summary, the results of this feasibility study essentially align with the existing literature on the effectiveness of interventions for foster parents. For instance, the meta-analysis by Schoemaker et al. [15] showed a significant reduction in the use of harsh parenting strategies (n = 4 studies), and in child behavioural problems (n = 33 studies), with a medium effect size (Hedges’ g).

### 5.1. Strengths and Limitations

This study was the first trial of *Fostering Changes* outside the UK and was conducted independently from the original *Fostering Changes* developers, which constitutes a strength. We were able to recruit carers of foster children aged 2–11 years with a balanced gender distribution for this study, which made it possible to obtain empirical results for a broad age band of children from kindergarten to school age. Thus, the current study contributes to addressing a research gap, as so far, no training for foster carers of children aged older than two years has been proven effective in Germany. Also, the outcomes for this trial were chosen carefully in order to ensure optimum comparability with previous studies on *Fostering Changes*. Finally, this study had a dropout rate below 10%.

All questionnaires employed in this trial were addressed to the foster parents as a source of information, which is a limitation, as additional external observers could have improved the quality of the data gathered. However, the inclusion of other stakeholders as further sources of information would have had created higher burdens for trial participation, as the consent of custodians would have been necessary (in Germany, foster carers often do not have legal parental responsibility for their foster children). Another limitation arises from four clustered datasets and the complete absence of follow-up results in three cases, despite efforts to address this issue by selecting appropriate statistical methods. Regarding further limitations, our study was a non-controlled trial; therefore, the observed changes cannot clearly be attributed to foster carers’ participation in the *Fostering Changes* parent training. Although in previous trials of *Fostering Changes*, the control groups made no improvement at all, so it is highly likely that the large improvements seen in this study are attributable to taking part in the intervention. Also, the sample size of 33 datasets analysed in this feasibility study is modest, which limits the generalisation of the results. In addition, *Fostering Changes* courses were offered only in Baden-Württemberg and were attended mainly by long-term and non-kinship foster families, which limits generalisability. Moreover, several foster carer and child characteristics were not assessed, meaning that we cannot determine how representative the sample is of foster families in Germany with regard to factors such as ethnic background or socio-economic status. Although we assessed other relevant child and family characteristics (e.g., foster children’s age and sex, or number of foster carers’ biological children), these unmeasured aspects constitute important limitations regarding the generalisability of the findings.

### 5.2. Conclusions

The results of this feasibility study, which is the first trial of *Fostering Changes* outside the UK, suggest that the German-translated version of *Fostering Changes* could be an effective intervention for foster carers and foster children, especially with regard to child behaviour and child psychopathology. As a next step, a randomised controlled trial of the German-translated *Fostering Changes* programme with a sample representative of the German foster care population should be considered in order to corroborate and extend the existing evidence on its effectiveness.

## Figures and Tables

**Table 1:** Effect Sizes of Previous Studies of *Fostering Changes*.

Variable	Moody et al., 2020 [26] (*N* = 312)			Briskman et al., 2010 [25] (*N* = 77)		Warman et al., 2006 [24] (*N* = 97)		Pallett et al., 2002 [22] (*N =* 36)	
	*d* ^a^	*d* ^b^	Measures	*d* ^b^	Measures	*d* ^c^	Measures	*d* ^c^	Measures
Problem behaviour (foster child)	-	-	CDPS	0.95	CDPS	1.23–1.36	CDPS	1.40	CDPS
						0.33	PSI (DC)	0.93	PSI (DC)
Psychopathology (foster child)	0.04	0.34	SDQ	0.30	SDQ	0.17	SDQ	0.06–0.29	SDQ
Quality of relationship (foster carer & child)	-	-	QARQ	0.40	QARQ				
Parenting style (foster carer)				-	APQ-SF	0.12	PSI (CCDI)	0.28	PSI (CCDI)
Coping strategies (foster carer)	0.15	0.30	CCS	0.50	CCS				
Self-efficacy (foster carer)	-	-	CEQ	0.70	CEQ				
Parental stress (foster carer)						0.21	PSI (CD)		

Note. *-* = Effect sizes as Cohen’s *d* were not reported in these publications. APQ-SF = Alabama Parenting Questionnaire—Short Form; CCS = Carer Coping Strategies; CDPS = *Carer-defined Problem Scale*; CEQ = *Carer Efficacy Questionnaire*; PSI = *Parenting Stress Index*, Subscales CD = *Carer Distress*, CCDI = *Carer–Child Dysfunctional Interaction*, DC = *Difficult Child*; QARQ = *Quality of Attachment Relationship Questionnaire*; SDQ = *Strengths and Difficulties Questionnaire*. ^a^ Effect sizes calculated based on the mean values of the intervention and control groups at the 12-month follow-up. ^b^ Effect sizes calculated based on the mean values of the intervention and control groups at the end of the course. ^c^ Effect sizes calculated based on the mean values of the same group measured at the beginning and the end of the course.

**Table 2:** Socio-demographic Characteristics of Foster Carers and Foster Children.

Foster Carers					
	***n***	**%**	***Md***	***IQR***	***Range***
Number of foster children in household			1	1	1–5
Number of own children in household			1	2	0–6
Duration of time as a foster carer (in months)			56	82.5	4–270
Number of children in care (including the current foster child)			2	1	1–27
Age (in years)			45	10.5	34–60
Sex					
male	6	18.8			
female	26	81.2			
Employment					
employed	18	56.3			
not employed	14	43.7			
Highest educational qualification					
Secondary school certificate (after 9th)	3	9.4			
Secondary school certificate (after 10th class)	14	43.7			
Entrance qualification for universities of applied sciences or universities	7	21.9			
Bachelor/Master/Diploma/PhD	8	25.0			
**Foster Children**					
	***n***	**%**	***Md***	***IQR***	***Range***
Age (in years)			5	6	2–11
Kinship care					
related to	1	3.2			
not related to	30	96.8			
Sex					
male	15	48.4			
female	16	51.6			
Education					
No external care	4	12.9			
Kindergarten	12	38.7			
Kindergarten for children with special needs	2	6.5			
Elementary School (until 4th class)	5	16.1			
School for children with special needs	6	19.4			
Secondary School (until 9th or 10th class)	1	3.2			
Secondary School (until 10th class)	1	3.2			
Type of placement					
Adoption	1	3.2			
Short-term care	1	3.2			
Long-term care	29	93.6			
Duration of current placement (in months)			40	50	4–135
Number of previous placements			1	1	0–3

**Table 3:** Results of Mixed Linear Models and Generalised Estimating Equations for Evaluating the effect of Time.

MLM—Variable: Time						
Outcome Variable	β	*df*	*p*	*T*	*95% CI*	
					LL	UL
Psychopathology (SDQ-total)	−1.40	49.56	0.003	−3.18	−2.28	−0.52
Quality of relationship (CRBI ratio)	0.26	36.17	<0.001	6.47	0.18	0.34
Parenting style (PS-S)	−0.30	61.42	<0.001	−5.89	−0.41	−0.20
Parental stress (PSS)	−1.35	51.79	0.058	−1.94	−2.74	0.05
**GEE—Variable: Time**						
**Outcome Variable**	**β**	***df***	***p***	***Wald-*** **χ^2^**	***95% Wald-CI***	
					**LL**	**UL**
Problem behaviours (CDPS)	−1.54	1	<0.001	−54.27	−1.99	−1.13
Quality of relationship (QUARQ)	0.77	1	<0.001	34.31	0.51	1.03

Note. CI = confidence interval; GEE = generalised estimating equations; LL = lower limit; MLM = mixed linear models; UL = upper limit. CDPS = *Carer-defined Problem Scale*; CRBI = *Child Relationship Behavior Inventory*; PSS = Parental Stress Scale; PS-S = *Parenting Scale*-short version; QUARQ = *Quality of Attachment Relationship Questionnaire*; SDQ = *Strengths and Difficulties Questionnaire—Total Difficulties*.

**Table 4:** Effect Sizes of the Change in Outcome Variables, by Time.

Variable	Time Points								
	t0–t1			t0–t2			t1–t2		
	|Cohen’s *d|*	*95% CI*		|Cohen’s *d|*	*95% CI*		|Cohen’s *d|*	*95% CI*	
		LL	UL		LL	UL		LL	UL
Child problem behaviour (CDPS)	1.87	1.29	2.43	1.92	1.31	2.52	0.09	−0.27	0.45
Child psychopathology (SDQ-total)	0.70	0.32	1.08	0.44	0.06	0.82	0.21	−0.16	0.57
Quality of relationship (CRBI ratio)	0.77	0.38	1.16	0.73	0.32	1.12	0.03	−0.33	0.39
Quality of relationship (QUARQ)	0.72	0.34	1.10	0.74	0.33	1.15	0.01	−0.35	0.36
Parenting style (PS-S total)	0.76	0.36	1.14	0.86	0.43	1.27	0.02	−0.34	0.38

Note. t0 = start of the course, t1 = end of the course, t2 = tree months after the end of the course. CI = confidence interval; LL = lower limit; UL = upper limit; CDPS = *Carer-defined Problem Scale*; CRBI = *Child Relationship Behavior Inventory*; PS-S = *Parenting Scale*-short version; QUARQ = *Quality of Attachment Relationship Questionnaire*; SDQ-total = *Strengths and Difficulties Questionnaire—Total Difficulties.* Effect sizes (Cohen’s *d*) were calculated using the average of the variances at both measurement points as the standardizer. Analyses included *n* = 33 for t0–t1 and *n* = 30 for t0–t2 and t1–t2 due to complete missing data at t2. Effect sizes (Cohen’s *d*) below 0.2 are considered as very small, from 0.2 to below 0.5 as small, from 0.5 to below 0.8 as moderate and from 0.8 as large. For better legibility, all effect sizes are reported as positive values. Direction of effects: t0–t1 and t0–t2: child problem behaviour, child psychopathology, and inappropriate parenting strategies: decrease; quality of relationship: increase. t1–t2: child problem behaviour: decrease, all other variables: increase.

## Data Availability

The quantitative data, given the small sample size, are not available, in order to protect the identity of participants.

## Supplementary Materials

The following supporting information can be downloaded at: https://www.mdpi.com/article/10.3390/children13010057/s1, Supplementary Table S1: Summary of Weekly Topics of a “Fostering Changes”-course; Supplementary Table S2: Mean Values, Standard Deviations and Ranges of the Outcome Variables, Across all Time Points; Supplementary Figure S1: Flowchart of Participation, Data Collection and Analysis; Supplementary Table S3: Utilisation of Public Support for the Target Foster Child Included in the Data Analysis. Supplementary Table S4: Interaction Effects of Mixed Linear Models and Generalised Estimating Equations for Variable Time and Covariates.

## Abbreviations

CDPS: Carer-defined Problem Scale; CRBI: Child Relationship Behavior Inventory; CRC: Child Relationship Checklist—Frequency Scale (part of CRBI); CRDQ: Child Relationship Development Questionnaire—Frequency Scale (part of CRBI); FASD: foetal alcohol spectrum disorder; GEE: generalised estimating equations; MCAR: missing completely at random; MLM: mixed linear models; ProQOL: Professional Quality of Life; PSS: Parental Stress Scale; PS-S: Parenting Scale-short version; QUARQ: Quality of Attachment Relationship Questionnaire; SDQ: Strengths and Difficulties Questionnaire; SDQ-total: Strengths and Difficulties Questionnaire—Total Difficulties; t0: assessment at the start of the course; t1: assessment at the end of the course; t2: assessment tree months after the end of the course; UK: United Kingdom.
