# Supplementary figures and images for: The “Fostering Changes” Parent Training Programme for Foster Carers: A Feasibility Study of the German Version
Source: Children (Basel). 2025 Dec 30;13(1):57. doi: 10.3390/children13010057 (PMC12840375; doi:10.3390/children13010057)

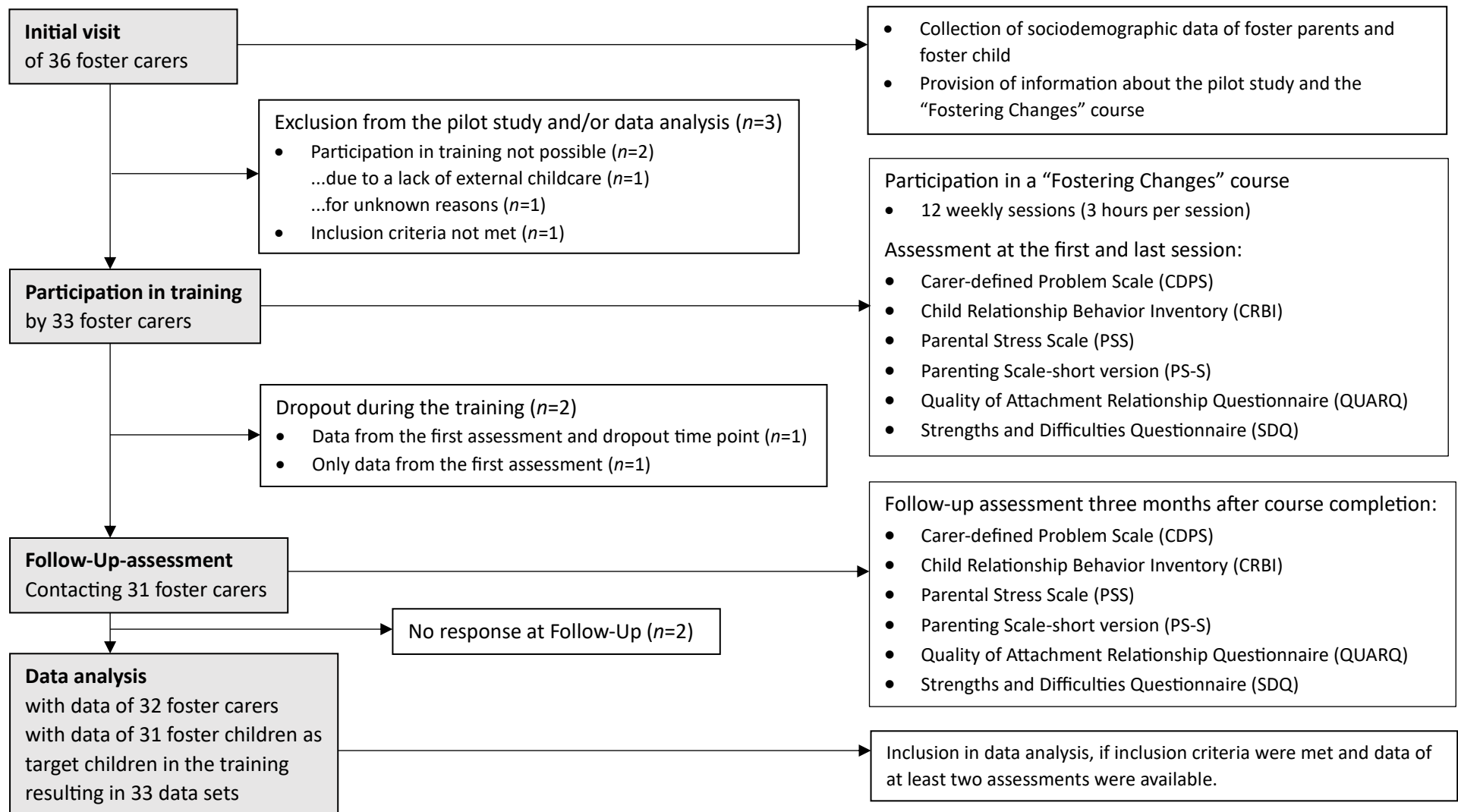

Supplement: Supplementary file 1 [file children-13-00057-s001.zip › Figure S1.pdf]
